# A Novel Kinetic Modeling of Enzymatic Hydrolysis of Sugarcane Bagasse Pretreated by Hydrothermal and Organosolv Processes

**DOI:** 10.3390/molecules28145617

**Published:** 2023-07-24

**Authors:** João Moreira Neto, Josiel Martins Costa, Antonio Bonomi, Aline Carvalho Costa

**Affiliations:** 1 Department of Engineering, Federal University of Lavras, Lavras 37200-000, MG, Brazil; 2 School of Food Engineering, University of Campinas, Campinas 13083-862, SP, Brazil; 3 Brazilian Biorenewables National Laboratory (LNBR), Brazilian Center for Research in Energy and Materials (CNPEM), Campinas 13083-100, SP, Brazil; 4 Laboratory of Fermentative and Enzymatic Process Engineering, School of Chemical Engineering, University of Campinas, Campinas 13083-852, SP, Brazil

**Keywords:** biomass, lignocellulose, parameter estimation, pretreatment, simulation

## Abstract

Lignocellulosic biomasses have a complex and compact structure, requiring physical and/or chemical pretreatments to produce glucose before hydrolysis. Mathematical modeling of enzymatic hydrolysis highlights the interactions between cellulases and cellulose, evaluating the factors contributing to reactor scale-up and conversion rates. Furthermore, this study evaluated the influence of two pretreatments (hydrothermal and organosolv) on the kinetics of enzymatic hydrolysis of sugarcane bagasse. The kinetic parameters of the model were estimated using the Pikaia genetic algorithm with data from the experimental profiles of cellulose, cellobiose, glucose, and xylose. The model considered the phenomenon of non-productive adsorption of cellulase on lignin and inhibition of cellulase by xylose. Moreover, it included the behavior of cellulase adsorption on the substrate throughout hydrolysis and kinetic equations for obtaining xylose from xylanase-catalyzed hydrolysis of xylan. The model for both pretreatments was experimentally validated with bagasse concentration at 10% *w*/*v*. The Plackett–Burman design identified 17 kinetic parameters as significant in the behavior of process variables. In this way, the modeling and parameter estimation methodology obtained a good fit from the experimental data and a more comprehensive model.

## 1. Introduction

The situation of world food production is linked to the problem of gases generated by the greenhouse effect, which creates pressure for the development of promising technologies for converting lignocellulosic biomass [1]. Its reuse aims to cogenerate energy and value-added products [2,3]. In addition, its use and recycling reduce environmental problems regarding the disposal of agro-industrial waste. One of the ways to process this residue is through enzymatic hydrolysis. The cellulase and xylanase enzymes hydrolyze cellulose and xylan into fermentable sugars such as glucose and xylose, respectively [4]. The technique is considered sustainable due to its low cost, ease of acquisition, and great potential for obtaining sugars from agro-industrial residues [5,6].

The structure and the composition of lignocellulosic biomass vary according to its source. Cellulose stores the energy conserved by photosynthesis and is one of the main components in lignocellulosic biomass. It is entangled in the hemicellulose structure and covered by lignin [7]. In biomass, cellulose is also found in the form of cellobiose, which has two glucose molecules [8]. For efficient conversion to obtain biofuels, cellulose polymers must be decomposed into small molecules, allowing microbial assimilation. Therefore, the outer layer of lignin needs to be broken down [9].

Sugarcane bagasse is an important byproduct with a high cellulose content [10,11]. It consists of cellulose (45%), hemicellulose (28%), and lignin (18%) [12]. Its pretreatment facilitates subsequent operations, which can result in second-generation ethanol [13]. Pretreatment can occur by various methods, such as physical–chemical, chemical, and biological [14]. The organosolv chemical method is a process of extracting lignin from lignocellulosic materials using organic solvents or an aqueous-organic solution. It can swell the plant cell walls and disrupt the lignin structure. The physicochemical hydrothermal method consists of keeping the lignocellulosic material in the presence of water over a wide range of 160–240 °C, facilitating enzymatic digestibility. It can efficiently convert hemicelluloses into soluble compounds that are mainly composed of mono- and oligosaccharides. However, lignin is not removed effectively [15,16].

Approximate simulations of the hydrolysis operation model the process to improve, optimize, and maximize the value derived from the biomass used as substrate [17]. A reliable hydrolysis model must reflect the actual process, considering the reaction rates, mechanisms, and activities of the reactants and products [18]. Substrate heterogeneity, enzyme inhibition, and operating conditions, such as pH and temperature control, make system modeling difficult.

Enzymatic hydrolysis models are classified according to the number of solubilization activities and substrate state variables [19]. They are known as non-mechanistic, semi-mechanistic, functional, and structural models. In semi-mechanistic models, equations for reaction rates are generally described using Michaelis–Menten enzymatic kinetics with or without incorporating enzyme adsorption effects, temperature, substrate characteristics (accessible surface area, crystallinity, etc.), and product-inhibitory effects. Although this model encompasses many industrial projects, it may be limited in the effect of substrate characteristics. Godoy et al. [20] considered a semi-mechanistic approach to modeling batch and semi-batch enzymatic hydrolysis of hydrothermal sugarcane bagasse. The fit demonstrated that the model performed well compared to the experimental data. For reactor design and process simulation and optimization, these models are crucial as they describe structural information.

In this regard, this study aimed to validate and develop the mathematical modeling of the kinetic parameters in the enzymatic hydrolysis of sugarcane bagasse subjected to hydrothermal and organosolv pretreatment. Relevant experimental data were systematically accumulated. The proposed semi-mechanistic model included the non-productive adsorption of cellulase on lignin and cellulase inhibition by xylose. Moreover, Plackett–Burman sensitive analysis was used to identify the significant parameters in the kinetic model of enzymatic hydrolysis.

## 2. Results and Discussion

### 2.1. Estimation of Kinetic Parameters for Sugarcane Bagasse under Hydrothermal and Organosolv Pretreatment

The model considered as input data the enzyme concentrations and the initial concentrations of cellulose, lignin, hemicelluloses (xylan), cellobiose, glucose, and xylose of each assay. The initial concentration of the resulting sugars in the hydrolysis (cellobiose, glucose, and xylose) was 0 mg/mL in all assays. Table 1 displays the cellulose, lignin, and xylan concentration in hydrothermal bagasse (HB) and organosolv bagasse (OB), while endoglycanase/cellobiohydrolase (EG/CBH), β-glucosidase (BG) concentration, and xylanase activity used in the model simulation are shown in Table 2. Supplementary Materials: Table S1 displays the main genetic parameters selected in the Pikaia genetic algorithm. As reported previously, the default values and options were adopted [21,22,23].

Table 3 shows the estimated kinetic parameters for HB and OB data compared to the other studies.

In addition to the different experimental conditions and substrates, the parameter difference may be related to assumptions in the development of the models. The hydrolysis phenomena not considered in the model may affect the parameter estimation step. For example, Zheng et al. [24] considered the effect of non-productive adsorption of lignin. However, the effect of xylose inhibition on enzymes was neglected. Flores-Sánchez et al. [27] added the formation and inhibition of arabinose in the model, while Prunescu and Sin [28] considered acetic acid formation and furfural inhibition. The model developed in this study, in addition to the phenomenon of non-productive adsorption of cellulase on lignin and inhibition of cellulase by xylose, included the behavior of cellulase adsorption on the substrate throughout the hydrolysis and kinetic equations for the formation of xylose from the hydrolysis of xylan catalyzed by xylanase.

Important differences in the order of magnitude, such as k_3iX_ and k_4iX_ in Table 3, demonstrate the contradictions and difficulty of direct comparison of model parameters. The comparisons can become inconvenient due to different experimental conditions, especially substrate and enzyme type, and operational factors, such as pH, temperature, and operation mode [29]. Differences in reaction rate constants can indicate biomass types and/or pretreatment method variations. These constants describe the susceptibility of the corresponding substrate in each of the reactions (r_1_, r_2_, and r_3_). Analyzing the constants k_1r_, k_2r_, and k_3r_ of HB and OB, the order of magnitude followed the same pattern, k_2r_ > k_1r_ > k_3r_. Therefore, in the hydrolysis of HB and OB, the homogeneous cellobiose hydrolysis reaction was faster than the heterogeneous reactions r_1_ and r_3_. The reaction rate constants k_1r_ and k_3r_ kept practically the same value in the hydrolysis of HB and OB. However, the reaction rate constant of cellobiose to glucose conversion (k_2r_) was higher in OB hydrolysis than in HB hydrolysis.

The enzymatic inhibition by each sugar is inversely proportional to its inhibition constant. The inhibition of the r_1_ reaction by cellobiose should be stronger than that by glucose because cellobiose is the product of the r_1_ reaction. On the other hand, since glucose is the direct product of the r_3_ reaction, this reaction is more strongly inhibited by glucose than by cellobiose. The xylose present in the r_1_, r_2_, and r_3_ reactions is not a direct product of the cellulase and β-glucosidase catalyzed reactions. Therefore, it acts as a weaker inhibitor than glucose and cellobiose, respectively. In general, the comparison between the inhibition constants is consistent in that K_1iX_ > K_1iG_ > $K_{1{\mathrm{iG}}_{2}}$ and K_3iX_ > $K_{2{\mathrm{iG}}_{2}}$ > K_3iG_ (Table 3).

The enzyme inhibition constants were different for the two pretreatments. The studied biomasses also presented different values of enzyme inhibition constant [24,25]. The oscillation observed in the behavior of the HB and OB inhibition constants made it difficult to establish a relationship between these effects and the type of biomass pretreatment. In r_1_, $K_{1{\mathrm{iG}}_{2}}$ decreased from 0.769 to 0.11 comparing HB with OB, indicating a greater inhibitory effect of cellobiose in converting cellulose to cellobiose in the hydrolysis of OB. However, in r_3_, $K_{3{\mathrm{iG}}_{2}}$ exhibited a stronger inhibitory effect on HB than on OB. The cellulase inhibition constant by glucose in r_1_ (K_1iG_) demonstrated a greater effect on HB than on OB. However, in r_3_, K_3iG_ exhibited a greater inhibitory effect on OB than on HB.

**Figure 1:**
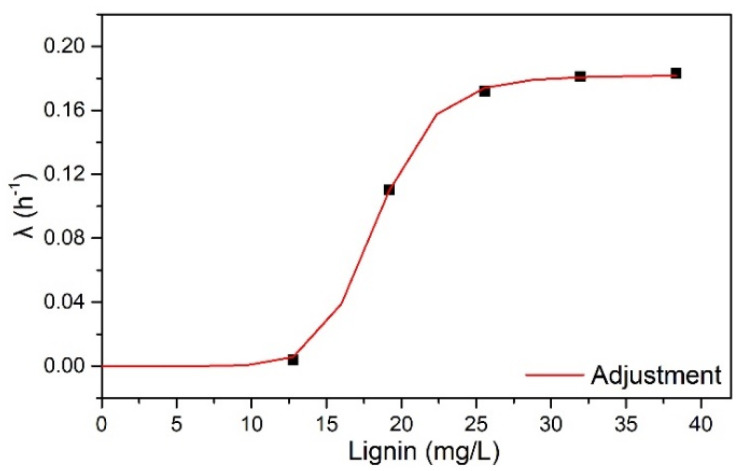
λ dependence with lignin concentration.

The parameter λ was dependent on the rate of decrease in cellulose surface area with the concentration of lignin (L) in HB, as illustrated in Figure 1. The increase in λ with increased lignin concentration was related to the decrease in glucose yield observed with increasing solids concentrations in enzymatic hydrolysis reactions. Lignin acted as a barrier that limited the availability of cellulose area accessible to enzyme action.

According to Figure 1, lignin exhibited a non-linear effect on λ, indicating two behaviors: (1) at low lignin concentrations (from 0 to 4% *w*/*v* HB), the λ profile was close to 0 and constant; (2) between concentrations of 12.8 to 38.4 mg/mL of lignin (from 4 to 12% m/v of HB), there was an exponential profile with a constant λ value from 25.6 mg/mL of lignin (8% m/v of HB).

The dependence of λ on L was fitted by a simple exponential function (Equation (1)), valid up to 40 mg/mL of lignin. The OriginPro 8.0 Software Levenberg–Marquardt algorithm (OriginLab, Northampton, MA, USA) estimated the following parameters: λ_max_—0.1817 h^−1^, n—9.45, k—18.354 h^−1^, and R^2^—0.999.

(1) $$\lambda =\frac{\lambda_{\max}L^{n}}{K^{n}{+L}^{n}}$$

As shown in Table 3, λ was not dependent on lignin concentration for OB. As a delignifying pretreatment, OB exhibited a lower lignin content (4.42%) than HB (31.97%), leading to a low lignin concentration in the enzymatic hydrolysis. The highest lignin concentration in the OB hydrolysis was 5.3 mg/mL (OB assay at 12% *w*/*v*).

**Figure 2:**
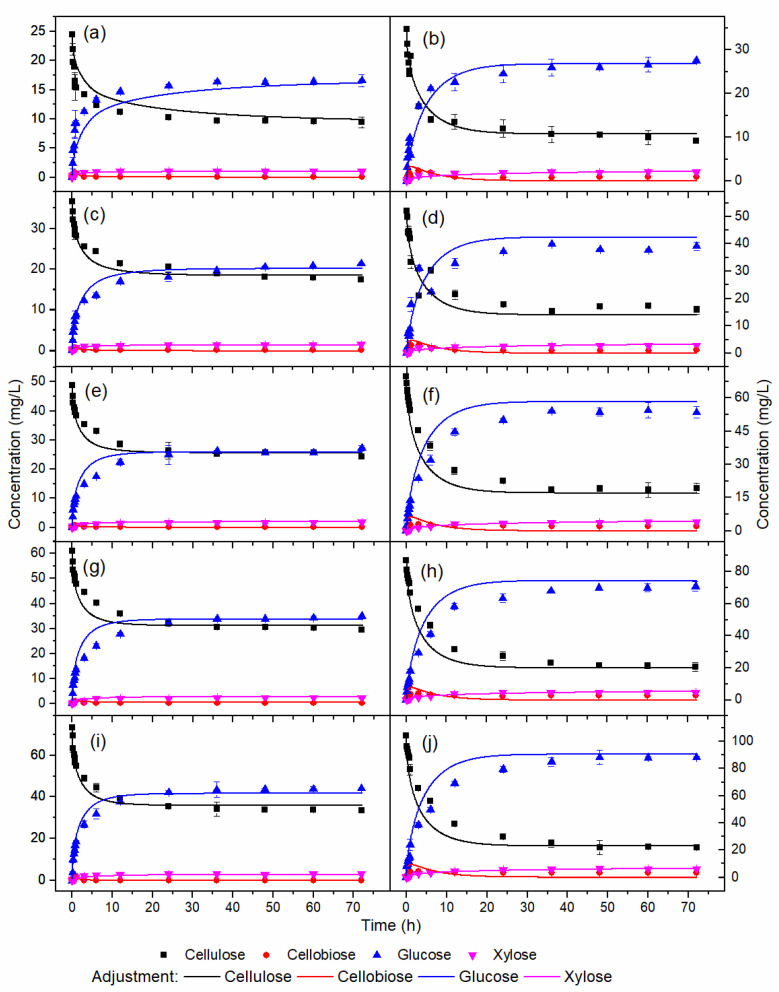
Temporal profiles of enzymatic hydrolysis with the following pretreatments and concentrations (% *w*/*v*): (**a**) HB 4, (**b**) OB 4, (**c**) HB 6, (**d**) OB 6, (**e**) HB 8, (**f**) OB 8, (**g**) HB 10, (**h**) OB 10, (**i**) HB 12, (**j**) OB 12.

**Figure 3:**
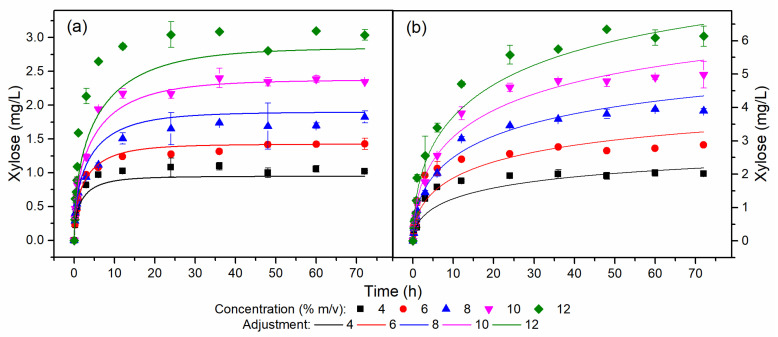
Xylose temporal profiles for (**a**) HB and (**b**) OB at different concentrations.

Figure 2 shows the simulation of enzymatic hydrolysis of HB and OB using the kinetic parameters of Table 3. The model represented the behavior of the experimental profiles of cellulose and glucose for the assays used in the parameter estimation procedure (Figure 2a–f,i,j), model validation (Figure 2g,h), and xylose temporal profiles (Figure 3). A semi-mechanistic model was a suitable choice for the enzymatic hydrolysis model. It presents the smallest possible number of parameters, reducing the amount of experimental data for estimating the values [30].

In Figure 2a, although the model represented the trend of the experimental profiles for the assay with 4% *w*/*v* HB in the initial hours of hydrolysis, it exhibited a simulated glucose profile slightly below the experimental profile. In addition, the simulated cellulose profile was slightly overestimated compared to the experimental.

In Figure 2c,e,g,i, similarly to Figure 2a, the model accurately described the experimental profiles of cellulose and glucose after 12 h of reaction. However, between the reaction times of 1 and 12 h, the simulated glucose profiles were slightly overestimated compared to the experimental. In addition, the cellulose profiles showed lower values than the experimental. Experimental/simulated cellulose and glucose profiles of HB and OB demonstrated rates of cellulose consumption and formation of similar glucose. All glucose was formed in the initial 15 h of hydrolysis. As previously reported, this observation was compatible since the reaction rate constants k_1r_ and k_3r_ for HB were close to the constants for OB.

Figure 3 displays the xylose temporal profiles for HB and OB. For a concentration of 12% *w*/*v* of HB, a simulated lower xylose profile and an overestimated profile were observed compared to the experimental data. The difference between the experimental and simulated data was due to the experimental profile of xylose corresponding to the 12% *w*/*v* HB assay presenting a conversion concerning xylan of 107%, due to an imprecision in the stage of quantification of the experimental data or even analysis of the composition of HB hemicelluloses. Furthermore, the content of hemicelluloses in HB was 2.1%, increasing the uncertainty in quantification. The conversion of xylan to xylose in HB was close to 100%, while the average conversion of xylan to xylose in OB was around 65%. The difference between the conversion values for each biomass was related to the xylan/xylanase ratio used in the assays. The xylan concentration in the OB was twice that of the HB, and as the enzyme dosage was the same in each biomass, the xylan/xylanase ratio in the OB was half that of the HB.

The residual standard deviation (RSD) of the kinetic model for the cellulose, glucose, and xylose concentrations of the assays for HB and OB are shown in Table S2. The cellulose, glucose, and xylose profiles for HB and OB showed RSD values close to just over 20%, demonstrating the excellent fit of the model to the experimental data. Although the glucose and xylose profiles exhibited RSD values of 22.98 and 21.44%, respectively, Figure 3 shows that the model followed the trend of the experimental data even without perfectly overlapping them. Therefore, the semi-mechanistic model described and predicted the enzyme–substrate conversion kinetics for engineering applications. Within the range of its validity (substrate loading range of 4 to 12% *w*/*v*), it assists in designing technologies for pilot- and industrial-scale and optimization studies [31,32].

### 2.2. Plackett–Burman Design for the Selection of Significant Parameters

A Plackett–Burman design was used to analyze the dynamic behavior of the 17 parameters of the kinetic model of enzymatic hydrolysis of HB. The responses to represent the batch experiment were the concentrations of C, G_2_, G, X_n_, and X at intervals from 10 min to 1 h, and at times 3, 6, 12, 24, 36, 48, 60, and 72 h. The kinetic parameters of the model analyzed were k_1r_, k_2r_, k_3r_, $K_{1{\mathrm{iG}}_{2}}$, K_1iG_, K_1iX_, K_2iG_, K_2m_, K_2iX_, $K_{3{\mathrm{iG}}_{2}}$, K_3iG_, K_3iX_, k_4_, K_eq_, K_4iX_, k_4s_, and λ. Values were evaluated at low (−) and high (+) levels. The difference between these levels and the nominal values of the parameters was 10%. The initial conditions of C, G_2_, G, X_n_, and X were set at 61.07, 0, 0, 2.1, and 0 mg/mL. The enzyme loads of cellulase, β-glucosidase, and xylanase enzymes were fixed at 15 FPU/g of HB (1.049 mg protein/mL), 25 CBU/g of HB (0.2543 mg of protein/mL), and 14.632 U/mL, respectively. The effects of kinetic parameters on responses were determined by BP factorial design using Statistica 7.0 Software (Statsoft).

**Figure 4:**
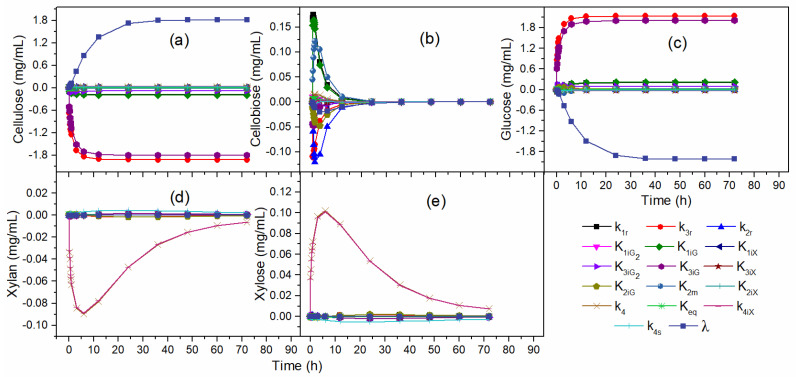
Effects of the kinetic parameters: (**a**) cellulose, (**b**) cellobiose, (**c**) glucose, (**d**) xylan, (**e**) xylose concentration.

Figure 4 illustrates the effects of the kinetic parameters over the time of hydrolysis. The parameter λ at the beginning of hydrolysis had a low effect on cellulose and glucose concentration, as shown in Figure 4a,c. However, over time the influence of λ became significant. The effects of all kinetic parameters for cellobiose concentration decreased with hydrolysis time (Figure 4b), demonstrating greater influence in the initial hours of reaction. Therefore, the analysis in a time interval of the hydrolysis can eliminate the procedure of re-estimating important parameters for describing the enzymatic hydrolysis. According to Figure 4d,e, k_4_ and k_4iX_ effects decreased along with the hydrolysis, tending to close to zero for the xylan and xylose concentration.

Table 4 lists the effects of kinetic parameters on C, G_2_, G, X_n_, and X concentrations. The black areas indicate that the parameter had a great influence on the response, the gray areas indicate low influence, and the white areas indicate practically negligible influence. The significant parameters for cellulose concentration were the same for glucose concentration. Therefore, the parameters with the greatest influence on cellulose and glucose concentration were k_3r_, K_3iG_, and λ. The parameters of low influence were k_1r_ and K_1iG_, while the parameters k_2r_, $K_{1{\mathrm{iG}}_{2}}$, K_1iX_, K_2iG_, K_2m_, K_2iX_, $K_{3{\mathrm{iG}}_{2}}$, K_3iX_, k_4_, K_eq_, K_4iX_, and k_4s_ showed no influence on the cellulose and glucose concentration in the hydrolysis.

For cellobiose concentration, the significant parameters were k_1r_, k_2r_, K_1iG_, and K_2m_, while k_3r_, $K_{1{\mathrm{iG}}_{2}}$, K_1iX_, K_2iG_, K_2iX_, $K_{3{\mathrm{iG}}_{2}}$, K_3iX_, k_4_, K_eq_, K_4iX_, k_4s_, and λ did not influence hydrolysis. The gradual reduction of all the effects of the parameters until null values at the end of the hydrolysis occurred due to the consumption of glucose to obtain glucose after the initial hours of hydrolysis, justifying the non-influence of the parameters in the final hours of reaction.

Significant parameters for xylan concentration were the same as for xylose concentration. The parameters k_4_ and K_4iX_ showed significant effects under xylan and xylose concentrations. The parameter k_4s_ indicated low influence, while the parameters k_1r_, k_2r_, k_3r_, $K_{1{\mathrm{iG}}_{2}}$, K_1iG_, K_1iX_, K_2iG_, K_2m_, K_2iX_, $K_{3{\mathrm{iG}}_{2}}$, K_3iG_, K_3iX_, λ, and K_eq_ did not influence the concentration of xylan and xylose.

## 3. Materials and Methods

### 3.1. Biomass Preparation and Pretreatment

Sugarcane bagasse (*Saccharum officinarum*) was dried at room temperature for 4 days, ground in a cutting mill (Pulverisette 19, Fritsch), and sieved with a 0.5 mm sieve and stored. Two pretreatments (hydrothermal and organosolv) were applied separately to the biomass. For the hydrothermal pretreatment, 300 g of dried bagasse and 3 L of distilled water were added to a 7.5 L reactor. The reaction occurred for 10 min at 190 °C. After pretreatment, the HB was washed with water until the pH remained constant to remove soluble compounds in the hydrolyzate.

For the organosolv pretreatment, 300 g of dried bagasse and 3 L of a water/ethanol solution (1:2 *v*/*v*) were added to a 7.5 L reactor. The reaction occurred for 150 min at 190 °C. The pretreated bagasse was washed with a 1% (*m*/*v*) sodium hydroxide solution to solubilize residual lignin from the fibers. After pretreatment, the OB was washed with water until the pH stabilized. Both HB and OB were dried at room temperature and stored.

### 3.2. Enzymatic Activity

The enzymes used were cellulase from *Trichoderma reesei* (Celluclast 1.5 L from Novozyme) and β-glycosidase from *Aspergillus niger* (Novozym 188). The cellulolytic activity was quantified in filter paper units per milliliter (FPU/mL). In addition, a 15 mmol/L cellobiose solution was used to determine β-glucosidase activity. Its unit was expressed in units per milliliter (CBU/mL). Cellulase indicated an enzymatic activity of 75.69 FPU/mL and β-glucosidase 491.71 CBU/mL.

Xylanase activity was determined by assays in 96-well conical-bottomed plates as here described: 10 μL of culture supernatant with a suitable dilution was added to 40 μL of 0.05 M sodium citrate buffer (pH 4.8) and 50 μL of 0.05% beechwood xylan substrate. The mixture was incubated in a thermocycler at 50 °C for 10 min. Then, the reducing sugar was measured by the 3,5-dinitrosalicylic acid method by adding 100 μL of reagent and incubated again in a thermocycler at 99 °C for 5 min with immediate cooling. After cooling, 100 μL was transferred to a 96-well Elisa plate where the absorbance reading at 540 nm was performed using a Spectra Plate Reader Max 384 (Molecular Device). The xylanase activity was 738.34 U/mL. One unit (U) of xylanase was defined as the amount of enzyme required to release 1 μmol of xylose per minute under the test conditions.

### 3.3. Enzymatic Hydrolysis

The enzymatic hydrolysis of the pretreated bagasse was performed in a 1 L reactor containing a mixture of 250 mL of citrate buffer with pH adjusted to 4.8 and supplemented with 0.02% sodium azide per gram of biomass. Different concentrations of pretreated bagasse were added to each assay (4, 6, 8, 10, and 12% *w*/*v*), and cellulase and β-glucosidase loads were fixed at 15 FPU/g of bagasse and 25 CBU/g of bagasse. The hydrolysis reaction occurred in a jacketed reactor at 50 °C with 150 rpm stirring. Aliquots were collected in duplicate at intervals of 10 min to 1 h. After 1 h, aliquots in duplicate were collected at times 3, 6, 12, 24, 36, 48, 60, and 72 h. All samples were boiled to deactivate the enzymes.

### 3.4. Quantification of Sugars

The boiled samples were filtered through a GS membrane filter in cellulose ester with 0.22 µm pores (Millipore), and the monosaccharide content (glucose, cellobiose, xylose, and arabinose) was quantified in an HPLC system (model 1260 Infinity Agilent Technologies HPLC) equipped with a refractive index detector. Separation was performed on an Aminex HPX-87H column at 35 °C using a 0.01 mol/L H_2_SO_4_ solution prepared with filtered and degassed ultra-pure water (Milli-Q) as mobile phase with a flow rate of 0.6 mL/min [33]. The compound separated in the stationary phase was monitored with a refractive index detector at 30 °C for a run time of 20 min.

### 3.5. Kinetic Model

**Figure 5:**
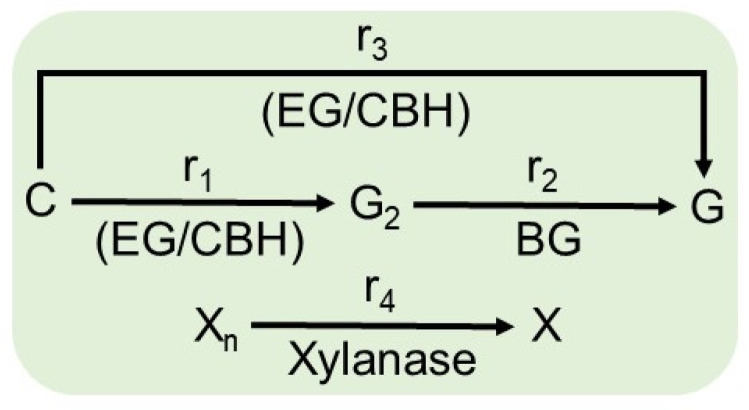
Mechanisms involved in the enzymatic hydrolysis of lignocellulosic biomass. r_1_ is the heterogeneous reaction rate for the production of cellobiose from cellulose catalyzed by the enzymes endoglycanase (EG) and cellobiohydrolase (CBH) adsorbed on cellulose; r_2_ is the homogeneous reaction rate for the production of glucose from cellobiose catalyzed by the enzyme β-glucosidase (BG) in solution; r_3_ is the heterogeneous reaction rate for the production of glucose from cellulose catalyzed by the enzymes EG and CBH adsorbed on cellulose; and r_4_ is the heterogeneous reaction rate for xylose production from xylan catalyzed by the enzyme xylanase adsorbed on xylan.

As shown in Figure 5, the hydrolysis of cellulose to glucose occurs by three reaction rates: r_1_, r_2_, and r_3_. r_1_ is the heterogeneous reaction rate for the production of cellobiose from cellulose catalyzed by the enzymes endoglycanase (EG) and cellobiohydrolase (CBH) adsorbed on cellulose; r_2_ is the homogeneous reaction rate for the production of glucose from cellobiose catalyzed by the enzyme β-glucosidase (BG) in solution; and r_3_ is the heterogeneous reaction rate for the production of glucose from cellulose catalyzed by the enzymes EG and CBH adsorbed on cellulose.

Each enzymatic reaction is potentially inhibited by the generated sugars (cellobiose and/or glucose) or other sugars present in the system, such as xylose. Although the enzymatic hydrolysis of cellulose does not result in xylose, it is present in the reaction medium in greater quantity, and its inhibitory effect on the catalytic action of cellulases has been reported [34]. Therefore, the r_4_ reaction, referring to the hydrolysis of xylan in xylose, was coupled to the model. r_4_ is the heterogeneous reaction rate for xylose production from xylan catalyzed by the enzyme xylanase adsorbed on xylan. Therefore, the model considered only the sugars generated by the reactions r_1_ to r_4_ (cellobiose, glucose, and xylose).

For the development of the model, the following considerations were addressed:

(1) The enzymatic adsorption follows the Langmuir adsorption isotherm, where the r_1_ and r_3_ reactions occur on the cellulose surface;
(2) Enzymatic deactivation by thermal and mechanical effects was negligible;
(3) The cellulosic matrix was uniform in terms of enzyme accessibility in the substrate, without distinction between the amorphous and crystalline fractions of cellulose;
(4) The cellulose consisted of EG, CBH, and low β-glucosidase activity. The model did not distinguish EG from CBH. Due to the low amount of β-glucosidase, the model considered the enzyme only from *Aspergillus niger*;
(5) The xylanase from the reaction medium was present in the cellulase used in the experimental assays;
(6) The hemicelluloses of HB and OB were composed solely of xylan;
(7) The conversion of cellobiose into glucose represented by r_2_ occurred in solution and followed the Michaelis–Menten kinetics;
(8) The conversion of xylan into xylose represented by r_4_ occurred in a single reaction, absent intermediate compounds such as xylobiose;
(9) The proportion of lignin exposed to the enzyme of the total lignin present in the pretreated bagasse was equal to 1, demonstrating that cellulose did not block the adsorption of enzymes on lignin [24];
(10) β-glucosidase did not adsorb to cellulose and lignin;
(11) The lignin of the pretreated biomass was not degraded during enzymatic hydrolysis.

Equations (2)–(8) describe the mass balance for the enzymatic hydrolysis model of HB and OB.

(2) $$\frac{\mathrm{dC}}{\mathrm{dt}}=-r_{1}-r_{3}$$

(3) $$\frac{{\mathrm{dG}}_{2}}{\mathrm{dt}}=1{.056r}_{1}-r_{2}$$

(4) $$\frac{\mathrm{dG}}{\mathrm{dt}}=1{.053r}_{2}+1{.111r}_{3}$$

(5) $$\frac{{\mathrm{dX}}_{n}}{\mathrm{dt}}=-r_{4}$$

(6) $$\frac{\mathrm{dX}}{\mathrm{dt}}=1{.136r}_{4}$$

(7) $$\mathrm{Cellulase}:\;E_{1T}{=E}_{1b}{+E}_{1f}$$

(8) $$\beta -\mathrm{glucosidase}:\;E_{2T}{=E}_{2b}{+E}_{2f}$$

where C, G_2_, G, X_n_, and X are the concentrations (mg/mL), t is the reaction time (h), and r_1_, r_2_, r_3_, and r_4_ are the reaction rates (mg/mL h). In Equations (3), (4) and (6), 1.056, 1.111, 1.053, and 1.136 are the stoichiometric factors of the reactions; 1.056 is the cellulose to cellobiose conversion factor, 1.111 is the cellulose to glucose conversion factor, 1.053 is the cellobiose to glucose conversion factor, and 1.136 is the xylan to xylose conversion factor.

The Langmuir isotherm for the adsorption of EG/CBH on HB and OB, which contains cellulose and lignin, is described by Equation (9) [33].

(9) $$E_{1b}=\frac{E_{\max}K_{p}E_{1f}}{{1+K}_{p}E_{1f}}S$$

where E_1b_ is the concentration of EG/CBH adsorbed on pretreated bagasse (mg protein/mL), E_max_ is the maximum amount of EG/CBH adsorbed per unit mass of pretreated bagasse (mg protein/g substrate), E_1f_ is the concentration of free EG/CBH in solution considering the pretreated bagasse as substrate (mg protein/mL), K_p_ is the dissociation constant for the adsorption/desorption reaction of EG/CBH with pretreated bagasse (mL/mg protein), and S is the pretreated bagasse concentration (mg/mL).

During the enzymatic hydrolysis of HB and OB, the adsorption of EG/CBH in the biomass decreases as the hydrolysis reaction proceeds. This behavior led to different profiles for the Langmuir isotherms and, therefore, different parameters in each isotherm. In this way, the E_max_ and K_p_ parameters were correlated with the reaction time through Equations (10) and (11).

(10) $$E_{\max}=e^{a+\mathrm{bX}+{\mathrm{cX}}^{2}}$$

(11) $$K_{p}=e^{a+\mathrm{bX}+{\mathrm{cX}}^{2}}$$

where a, b, and c are constants and X is the conversion (%). Values of a, b, and c are displayed in Table 5, as adjusted in a previous study [35].

Equation (12) describes the Langmuir adsorption isotherm of cellulase on lignin.

(12) $$E_{1\mathrm{bL}}=\frac{E_{\mathrm{maxL}}K_{\mathrm{pL}}E_{1\mathrm{fL}}}{{1+K}_{\mathrm{pL}}E_{1\mathrm{fL}}}L$$

where E_1bL_ is the concentration of EG/CBH adsorbed on lignin (mg protein/mL), E_maxL_ is the maximum amount of EG/CBH adsorbed per unit mass of lignin (mg protein/g lignin), E_1fL_ is the concentration of EG/CBH free in solution considering lignin as substrate (mg protein/mL), K_pL_ is the dissociation constant for the adsorption/desorption reaction of EG/CBH in lignin (mL/mg protein), and L is the lignin concentration (mg/mL).

As the adsorption of EG/CBH occurs on both cellulose and lignin, the amount of EG/CBH adsorbed on cellulose, E_1bC_, is calculated by Equation (13).

(13) $$E_{1\mathrm{bC}}=E_{1b}-E_{1\mathrm{bL}}$$

where E_1bC_ is the concentration of EG/CBH adsorbed on pretreated bagasse cellulose (mg protein/mL).

The kinetic rates described in Figure 5 are estimated according to Equations (14)–(17).

(14) $$r_{1}=\frac{k_{1r}E_{1\mathrm{bc}}C}{1+\frac{G_{2}}{K_{{1\mathrm{iG}}_{2}}}+\frac{G}{K_{1\mathrm{iG}}}+\frac{X}{K_{1\mathrm{iX}}}}e^{-\lambda t}$$

(15) $$r_{2}=\frac{k_{2r}E_{2f}G_{2}}{K_{2m}\left(1+\frac{G}{K_{2\mathrm{iG}}}+\frac{X}{K_{2\mathrm{iX}}}\right)+G_{2}}$$

(16) $$r_{3}=\frac{k_{3r}E_{1\mathrm{bc}}C}{1+\frac{G_{2}}{K_{{3\mathrm{iG}}_{2}}}+\frac{G}{K_{3\mathrm{iG}}}+\frac{X}{K_{3\mathrm{iX}}}}e^{-\lambda t}$$

where k_ir_ are reaction rate constants (i = 1 for reaction of cellulose to cellobiose (mL/mg h); i = 2 for reaction of cellobiose to glucose (mL/mg h); and i = 3 for reaction of cellulose to glucose (h^−1^). $K_{1{\mathrm{iG}}_{2}}$, K_1iG_, and K_1iX_ are competitive inhibition constants of EG/CBH by cellobiose, glucose, and xylose in r_1_ (mg/mL). K_2iG_ and K_2iX_ are competitive inhibition constants of β-glucosidase by glucose and xylose in r_2_ (mg/mL). $K_{3{\mathrm{iG}}_{2}}$, K_3iG_, and K_3iX_ are competitive inhibition constants of EG/CBH by cellobiose, glucose, and xylose in r_3_ (mg/mL). K_2m_ is the cellobiose saturation constant for β-glucosidase (mg/mL), E_2f_ is the concentration of free β-glucosidase in solution (mg/mL), and λ is the rate of decrease in cellulose surface area (h^−1^).

The xylan reaction rate equation, r_4_, considered xylan (X_n_) a limiting term for xylose formation.

(17) $$r_{4}=\frac{K_{4}X_{n}}{1+\frac{X}{K_{4\mathrm{iX}}}}\frac{X_{n}}{k_{S}{+X}_{n}}$$

where K_4_ is a concentrated constant of the reaction rate of xylan in xylose with dependence on the xylanase dosage (h^−1^), K_4iX_ is the competitive inhibition constant of xylanase per xylose (mg/mL), and k_S_ is the saturation constant for the term of substrate limitation (mg/mL). An analogous Langmuir-type dependence between the xylanase activity in the reaction medium and the K_4_ constant was described by Equation (18), as reported previously [36].

(18) $$r_{4}=\frac{k_{4}E_{3}}{K_{\mathrm{eq}}{+E}_{3}}$$

where E_3_ is the activity of the xylanase enzyme (U/mL), k_4_ is the maximum specific rate of hydrolysis of xylan to xylose (h^−1^), and K_eq_ is the saturation constant for adsorption of the xylanase enzyme (U/mL).

### 3.6. Parameter Estimation

Compaq Visual Fortran software version 6.6 estimated and modeled the parameters. The model’s resolution (Equations (2)–(6)) was performed in Fortran language with an integration algorithm based on the fourth-order Runge–Kutta method (IVPRK routine from the IMSL Math Library Fortran-90). The kinetic parameters of the model (k_1r_, k_2r_, k_3r_, $K_{1{\mathrm{iG}}_{2}}$, K_1iG_, K_1iX_, K_2iG_, K_2iX_, K_2m_, $K_{3{\mathrm{iG}}_{2}}$, K_3iG_, K_3iX_, λ, k_4_, K_eq_, K_4iX_, and k_S_) were estimated using the Pikaia genetic algorithm. This optimization subroutine implemented in Fortran estimated the parameters through the minimization of an objective function E(θ), according to Equation (19). θ is the vector that contains all the kinetic parameters to be optimized. The objective of the optimization is to find θ by minimizing the objective function using the experimental profiles of cellulose, cellobiose, and glucose in the bagasse concentration range defined in Section 3.3.

(19) $$E\left(\theta \right)=\sum_{i=1}^{\mathrm{np}}\sum_{i=1}^{m}\left[{\left(\frac{C_{i,j}{-\mathrm{Ce}}_{i,j}}{{\mathrm{Ce}}_{i\;\max}}\right)}^{2}+{\left(\frac{G_{2i,j}{-G}_{2}e_{i,j}}{G_{2}e_{i\;\max}}\right)}^{2}+{\left(\frac{G_{i,j}{-\mathrm{Ge}}_{i,j}}{{\mathrm{Ge}}_{i\;\max}}\right)}^{2}+{\left(\frac{X_{i,j}{-\mathrm{Xe}}_{i,j}}{{\mathrm{Xe}}_{i\;\max}}\right)}^{2}\right]$$

In Equation (19), np is the number of points referring to batch enzymatic hydrolysis samples, and m is the number of experimental profiles. C_ei,j_, G_2_e_i,j_, Ge_i,j_, and Xe_i,j_ are the concentrations of cellulose, cellobiose, glucose, and xylose measured at sampling time i for profile j. C_i,j_, G_2i,j_, G_i,j_, and X_i,j_ are the concentrations of cellulose, cellobiose, glucose, and xylose predicted by the model at sampling time i for profile j. Ce_imax_, G_2_e_imax_, Ge_imax_, and Xe_imax_ are the maximum cellulose, cellobiose, glucose, and xylose concentrations measured at sampling time i for profile j. Four profiles of cellulose, cellobiose, glucose, and xylose obtained from the enzymatic hydrolysis of HB and OB were used to fit the kinetic model. The validation of the model considered the profiles at a concentration of 10% *m*/*v*.

## 4. Conclusions

A semi-mechanistic model for the enzymatic hydrolysis processes of HB and OB was successfully developed and validated. The model structure considered the representation of the cellulase/cellulose system, the inhibition by the sugars released by the biomass (cellobiose, glucose, and xylose), and enzymatic adsorption (productive and non-productive adsorption of enzymes on cellulose and lignin). The model adequately described the concentration of cellulose, glucose, and xylose and initial cellulase and β-glucosidase. However, the kinetic parameters estimated for HB and OB were different, demonstrating the influence of pretreatment on the morphological characteristics and composition of the biomass in enzymatic hydrolysis. This model differed from previous models. It included the behavior of cellulase adsorption on the substrate and kinetic equations for xylose formation. The Plackett–Burman design indicated that some parameters influenced the beginning of hydrolysis while others influenced the end. Therefore, this tool was important for determining the significant parameters of the model and thus eliminating the re-estimation procedure.

## Figures and Tables

**Table 1:** Concentration of cellulose, lignin, and xylan in HB and OB.

Pretreatment	Bagasse Concentration % (m/v)	Cellulose (mg/mL)	Lignin (mg/mL)	Xylan (mg/mL)
HB	4	24.4 ± 3.9	12.79 ± 0.02	0.84 ± 0.02
HB	6	36.6 ± 5.8	19.18 ± 0.03	1.26 ± 0.04
HB	8	48.9 ± 7.8	25.58 ± 0.04	1.68 ± 0.05
HB	10	61.1 ± 9.7	31.97 ± 0.05	2.10 ± 0.06
HB	12	73.3 ± 11.6	38.36 ± 0.06	2.52 ± 0.07
OB	4	34.8 ± 1.6	2.7 ± 1.1	1.8 ± 0.1
OB	6	52.2 ± 2.4	4 ± 2	2.65 ± 0.15
OB	8	69.5 ± 3.2	5.3 ± 2.2	3.5 ± 0.2
OB	10	87 ± 4	6.6 ± 2.7	4.42 ± 0.25
OB	12	104.3 ± 4.8	8 ± 3	5.3 ± 0.3

**Table 2:** EG/CBH, BG concentration, and xylanase activity for HB and OB.

Bagasse Concentration % (m/v)	EG/CBH (mg/mL)	BG (mg/mL)	Xylanase (U/mL)
4	0.420	0.1017	5.853
6	0.629	0.1526	8.779
8	0.839	0.2034	11.706
10	1.049	0.2543	14.632
12	1.259	0.3052	17.559

**Table 3:** Estimated kinetic parameters of the Pikaia genetic algorithm for HB and OB data.

	Current Study		[24]	[25]	[26]	[27]	[28]
Parameter	HB	OB	Acid Treatment—Wild Ryegrass	Cotton Pretreated with N-Oxide-N-Methylmorpholine	Sugarcane Straw	Corncob Stock	Wheat Straw
k_1r_ (mL/mg h)	19.178	20.289	16.5	32.10	0.509	94.72	1.224
k_2r_ (h^−1^)	196.56	230.82	267.6	263.89	165.7	432.16	252
k_3r_ (mL/mg h)	8.576	7.236	7.1	13.56	12.75	958.3	19.08
K_1iG2_ (mg/mL)	0.769	0.11	0.02	7.52	0.016	1.00 × 10^−5^	0.0014
K_1iG_ (mg/mL)	0.03	4.875	0.1	0.34	0.710	7.33	0.073
K_1iX_ (mg/mL)	31.92	285.15	-	-	0.559	8.92	0.1007
K_2iG_ (mg/mL)	14.853	10.02	2.1	3.19	0.011	1.45 × 10^−5^	3.9
K_2m_ (mg/mL)	22.48	11.295	25.5	11.63	47.20	0.022	24.3
K_2iX_ (mg/mL)	278.2	51.48	-	-	110.0	39.19	201
K_3iG2_ (mg/mL)	0.913	2.574	132.5	38.41	89.18	7.33	132
K_3iG_ (mg/mL)	0.853	0.167	0.01	1.58	0.551	1.15 × 10^−3^	0.34
K_3iX_ (mg/mL)	86.38	180.22	-	-	0.581	6.13	0.029
k_4_ (h^−1^)	18.066	37.56	-	-	13.46 ^b^	167.27 ^b^	9.72 ^b^
Keq (U/mL)	0.0786	0.0066	-	-	-	-	-
K_4iX_ (mg/mL)	0.0111	0.0262	-	-	134.1	23.12	201
ks (mg/mL)	0.0354	45.8	-	-	-	-	-
^a^ λ (h^−1^)	$\frac{0.1817L^{9.45}}{18.3549^{9.45}+L^{9.45}}$	0.2004	-	-	-	-	-

^a^ Function of λ dependence on lignin concentration at concentrations from 0 to 12% *w*/*v*. ^b^ k_4_ in: (mL/mg h).

**Table 4:** Effect of kinetic parameters on C, G_2_, G, X_n_, and X concentrations during hydrolysis.

Parameter	Cellulose	Cellobiose	Glucose	Xylan	Xylose
k_1r_					
k_2r_					
k_3r_					
K_1iG2_					
K_1iG_					
K_1iX_					
K_2iG_					
K_2m_					
K_2iX_					
K_3iG2_					
K_3iG_					
K_3iX_					
k_4_					
K_eq_					
K_4iX_					
k_4s_					
λ					

**Table 5:** Values of constants and determination coefficient (R^2^) for Langmuir isotherm.

Parameters	a	b	C	R^2^
E_max_ (HB)	3.607	−0.00719	−0.0000772	0.980
K_p_ (HB)	0.2501	0.00134	−0.000146	0.979
E_max_ (OB)	3.383	−0.0027	0.000003	0.996
K_p_ (OB)	1.008	−0.014	0.00008	0.889

## Data Availability

The data presented in this study are available in the article.

## Supplementary Materials

The following supporting information can be downloaded at: https://www.mdpi.com/article/10.3390/molecules28145617/s1, Table S1. Technical settings selected in the Pikaia genetic algorithm; Table S2. Residual standard deviation for the kinetic model prediction of both pretreated bagasse.
