# Supplementary material for: A Novel Kinetic Modeling of Enzymatic Hydrolysis of Sugarcane Bagasse Pretreated by Hydrothermal and Organosolv Processes
Source: Molecules. 2023 Jul 24;28(14):5617. doi: 10.3390/molecules28145617 (PMC10386732; doi:10.3390/molecules28145617)
Supplement: Supplementary file 1 [file molecules-28-05617-s001.zip › molecules-2502764-supplementary.pdf]

A novel kinetic modeling of enzymatic hydrolysis of sugarcane bagasse pretreated by the hydrothermal and organosolv process

João Moreira Neto [1,*], Josiel Martins Costa [2,*], Antonio Bonomi [3] and Aline Carvalho Costa [4]

[1] Department of Engineering, Federal University of Lavras, Lavras, 37200-000, MG, Brazil
[2] School of Food Engineering, University of Campinas, Monteiro Lobato St., 80, Campinas-SP, 13083-862, Brazil
[3] Brazilian Biorenewables National Laboratory (LNBR), Brazilian Center for Research in Energy and Materials (CNPEM), Campinas, 13083-100, SP, Brazil
[4] Laboratory of Fermentative and Enzymatic Process Engineering, Albert Einstein Ave., 500, Campinas, 13083-852, SP, Brazil
* Correspondence author: joao.neto@ufla.br, josiel@unicamp.br

Table S1. Technical settings selected in the Pikaia genetic algorithm.

Chosen option	Parameters	Parameters value
Elitism, variable mutation, breeding plan: complete generational replacement	Number of chromosome segments	17
	Population size	100
	Crossover probability	0.85
	Initital mutation rate	0.005
	Minimum mutation rate	0.0005
	Maximum mutation rate	0.25
	Number of generations	300

Table S2. Residual standard deviation for the kinetic model prediction of both pretreated bagasse.

Pretreatment	Bagasse concentration %(m/v)	Residual standard deviation (%)		
		Cellulose	Glucose	Xylose
HB	4	17.06	22.11	16.16
	6	4.92	10.08	6.28
	8	6.21	13.65	13.03
	10	7.59	16.47	7.37
	12	5.73	10.98	20.73
	15	9.84	22.98	15.23
OB	4	11.85	14.90	21.44
	6	12.73	16.77	21.23
	8	12.69	13.60	10.41
	10	13.57	14.29	11.11
	12	15.10	14.11	12.11
